# Does kinship with the silverback matter? Intragroup social relationships of immature wild western lowland gorillas after social upheaval

**DOI:** 10.1007/s10329-024-01149-1

**Published:** 2024-08-10

**Authors:** Masaya Tamura, Etienne François Akomo-Okoue, Lilian Brice Mangama-Koumba, Ebang Ella Ghislain Wilfried, Fred Loïc Mindonga-Nguelet

**Affiliations:** 1 Laboratory of Human Evolution Studies, Graduate School of Science, Kyoto University, Kitashirakawa-Oiwake-cho, Sakyo-ku, Kyoto, 606-8502 Japan; 2 Institut de Recherche en Ecologie Tropicale (IRET/CENAREST), BP 13354, Libreville, Gabon

**Keywords:** Familiarity, Kinship, One-male group, Social relationship, Western lowland gorilla

## Abstract

In primates living in one-male groups, the sole resident male is often an important social partner for group immatures. For such groups, however, replacement of the male and subsequent disruptions of their relationships are almost inevitable. Here, we described social relationships of immature wild western lowland gorillas within a habituated group, where two natal and eight immigrant immatures lived with the resident silverback. We recorded 5 m proximities among group members as an indicator of social closeness. We found that natal immatures spent more time within 5 m of the silverback than immigrant ones. The social closeness between the silverback and the younger immigrant immatures sharply increased after 1 year, but these values were still below those of the natal immatures. Regarding the development of independence from the mother, we found no significant difference between natal and immigrant immatures. The socially preferred nonmother mature for natal immatures was the silverback, whereas many immigrant immatures preferred a paternal adult sister who had previously co-resided with them in a previous group. Our results suggest that familiarity may be an important determinant of the social closeness between the silverback and immatures, but 1 year of co-residence might be too short to construct sufficient familiarity. The paternal sister may have played a pivotal role in the assimilation of immigrant immatures into the non-natal group. Nonetheless, it is not negligible that the silverback and immigrant immatures formed day-to-day close proximities. His tolerance toward co-residence with immigrant immatures can be considered a reproductive tactic.

**Supplementary Information:**

The online version contains supplementary material available at 10.1007/s10329-024-01149-1.

## Introduction

In primates living in pair-bonded or one-male groups, a sole resident male is often an important social partner for group immatures. In such species, these social relationships are normally characterized by affiliative stemming from their high paternity certainty (Huck and Fernandez-Duque 2012a; Smuts and Gubernick 1992; van Schaik and Paul 1996). Further, some males in such social groups exhibit various direct caring behaviors toward immatures, including grooming, carrying, food sharing, playing, and protecting (Kleiman and Malcolm 1981). The establishment of an affiliative relationship with the resident male is thought to provide fitness benefits to group immatures (Clutton-Brock 2016; Huck and Fernandez-Duque 2012a).

However, in such species, the replacement of the resident male due to death or takeover is common (Teichroeb and Jack 2017). It can be expected that social relationships between a replaced male and remaining immatures would collapse. Once a replacement occurs, the remaining immatures are forced to meet a new male. Usually, a new male shows hostility toward the remaining immatures owing to unrelatedness; consequently, dependent infants are sometimes killed (Palombit 2012, 2015), and weaned immature males are evicted from the group (Ohsawa 2003; Steenbeek 1996; Steenbeek et al. 2000). Nevertheless, in some species, it has been documented that immatures withstood this unfavorable situation through behavioral modification and reconstructing social relationships. For instance, immatures of pair-bonding equatorial sakis (*Pithecia aequatorialis*) increased proximity with their mother while avoiding the new male (Di Fiore et al. 2007), older siblings of monogamous owl monkeys (*Aotus* spp.) provided additional care for the remaining infant, instead taking the role of the missing father (Fernandez-Duque et al. 2008; Jantschke et al. 1998), and young langurs (*Semnopithecus* spp.) living in a one-male group rapidly peripheralized on the advent of a new adult male (Boggess 1980; Rudran 1973). Investigating how immatures cope with the loss of resident males may lead to a better understanding of how pair-bonded or one-male social systems can be maintained despite the inevitability of group disintegration.

The species of the genus *Gorilla* evolved in a one-male polygynous social system, judging from their morphology; they show significant sexual dimorphism in body and canine size, and the males have small testes (Harcourt and Stewart 2007). Adult male gorillas, called silverbacks, are famous for exhibiting high affiliation toward group immatures (Smuts and Gubernick 1992). As seen in most primate species, the mother is the most important social partner for immature gorillas during their early infancy (Fletcher 2001). However, they gradually shift their social focus from the mother to the resident silverback around their age of weaning (Harcourt 1979; Rosenbaum et al. 2011; Stewart 2001). Most immatures (0–8 years old) spend more time near the silverback than they do near non-mother adult females and often cluster around him to play (Stewart 2001). Furthermore, the silverback shows high tolerance for being treated like playground equipment (Fletcher 1994; Fossey 1979; Rosenbaum and Silk 2022; Schaller 1963). Direct play, carrying, grooming, holding, and resting in contact are also documented as affiliative interactions between resident silverbacks and group immatures (Rosenbaum et al. 2015). Meanwhile, paternal loss during the first year of life is associated with high mortality in early life (Morrison et al. 2023; Robbins et al. 2013). Thus, the resident silverback is an important social partner for immature gorillas, and his presence has a significant impact on immatures’ fitness.

Gorillas living in one-male groups face a risk of group disintegration due to the death of the resident silverback. Because female gorillas are normally not philopatric (Robbins and Robbins 2018), each of the remaining females (with their offspring) subsequently will meet and join a new silverback in a process referred to as involuntary transfer (Robbins 1995; Robbins et al. 2004; Stokes et al. 2003). If a female already has an unweaned infant when joining a new silverback, infanticide of this offspring is not unusual (Caillaud et al. 2020; Watts 1989; Yamagiwa et al. 2009). One previous report found that one infant male survived an involuntary transfer but had been subjected to an intensive attack by the new silverback (Sicotte 2000). Weaned immature males join non-reproductive groups or become solitary instead of transferring to a reproductive group (Robbins 1995, 2001; Robbins and Robbins 2018; Yamagiwa 1987; but see Robbins et al. 2019). Immatures experiencing group disintegration due to the loss of a resident silverback are expected to have difficulty establishing an alternative affiliative relationship with a new silverback.

Mountain gorillas (*Gorilla beringei beringei*) form multi-male groups, potentially as a counterstrategy to avoid the negative consequences of group disintegration caused by the loss of the resident silverback. About 40% of reproductive groups in this subspecies have multiple silverbacks (Kalpers et al. 2003; Robbins et al. 2009). These groups avoid disintegration because a subordinate male inherits the group following the death of the dominant silverback (Robbins 2001, but see Caillaud et al. 2020). Further, in multi-male groups, the social relationships of young gorillas with resident silverbacks are flexible. They normally form close ties with a dominant silverback during their life (Rosenbaum et al. 2015). However, if immatures lose the silverback, they re-establish alternative ties with other silverbacks in the same group (Rosenbaum et al. 2016a). Long-term studies featuring detailed behavioral observations showed that mountain gorilla societies have flexible social structures and intragroup social relationships enabling immatures to compensate for the loss of a silverback.

By contrast to the case of mountain gorillas, one-male groups are predominant (~95%) in all reported populations of wild western lowland gorillas (*G. gorilla gorilla*) (Gatti et al. 2004; Magliocca et al. 1999; Parnell 2002; Robbins et al. 2004). Due to the prevalence of the one-male group, relative to mountain gorillas, higher rates of group disintegration and subsequent involuntary transfers are expected (Robbins et al. 2004). In fact, at Mbeli Bai, Republic of Congo, multiple cases of group disintegrations have been recorded, and 11 infants disappeared following the disintegration of the natal group, in inferred infanticide (Manguette et al. 2020). Nevertheless, some infants have survived involuntary transfers and consequently co-reside with the new silverback (Manguette et al. 2020; Robbins et al. 2004; Stokes et al. 2003). Further, involuntary transfers to non-natal groups by weaned immatures from juveniles to young silverbacks have also been documented (Robbins et al. 2004). Additionally, several studies have provided genetic evidence to suggest the co-residences of a silverback and unrelated immatures in the same groups (Arandjelovic et al. 2010, 2014; Douadi et al. 2007; Forcina et al. 2019; Hagemann et al. 2018; Masi et al. 2021). These studies imply that in western lowland gorilla societies, mechanisms exist enabling immatures to co-reside with nonfather silverbacks within the same group. However, no detailed information has been documented regarding the social relationships of immature western lowland gorillas within non-natal groups, primarily due to difficulties in group habituations and direct observations in a dense forest habitat (Harcourt and Stewart 2007; Robbins and Robbins 2018).

In Moukalaba-Doudou National Park, Gabon, two one-male groups of western lowland gorillas disintegrated between 2016 and 2018, likely due to the death of the resident silverback. Following this, eight immatures (2–8 years old) immigrated to an adjacent one-male group that already had two offspring. Thus, a habituated one-male group was formed where both related and unrelated immatures to the resident silverback lived simultaneously. Taking advantage of this interesting situation, we provide the first detailed description of the social relationships of immature western lowland gorillas living in a non-natal group with an unrelated silverback.

Using these samples, however, we cannot truly separate out the effect of relatedness from familiarity with the silverback. This is because the two related immatures did indeed spend far more time around the silverback than the unrelated immatures who joined the group later in their lives. In other words, the related immatures were necessarily much more familiar with the silverback than the unrelated ones in our study condition. Then, afterward, we referred to the related immatures to the silverback as “natal immature(s),” and we called the unrelated immatures from the disintegrated groups “immigrant immature(s).”

In this study, we first discuss the relative effects of kinship and familiarity with the resident silverback to determine differences in intragroup social relationships between natal and immigrant immatures. Furthermore, in our observations and analyses, we noticed that the presence of an adult female named *Randa* might be an important figure for immigrant immatures. We focused on this adult female’s function in the assimilation of immigrant immatures into the non-natal group. Finally, we discuss the tolerance of the silverback toward immigrant immatures with respect to males’ reproductive tactics.

## Methods

### Study site

We conducted this study in Moukalaba-Doudou National Park, Gabon. It is located in the southwest of Gabon and has an area of 5,028 km^2^, consisting of a mosaic of forest, savanna, and swamp. Our research area (~30 km^2^) falls in the northeastern part of the park. More details on this site can be found in Takenoshita et al. (2008). In Moukalaba, long-term socio-ecological studies of wild western lowland gorillas have been ongoing since 2001, focusing on habituated groups (Ando et al. 2008).

### Subject group

The study subject was a habituated one-male group of wild western lowland gorillas, termed the Nidai Group (Table 1). This group is presumed to have five members prior to the social upheaval described below, including one silverback (*Nidai*), two adult females (*Randa* and *Ngou*), and their infants (*Ranguisa* and *Prince*). Between March and September 2018, the group experienced mass immigration from two neighboring groups, the Gentil Group and the Martial Group, due to their disintegration (Takenoshita et al., in prep; Tamura et al., in prep). At that point, 15 individuals immigrated into the Nidai Group, including three adult females, one young silverback, three blackbacks, three subadults, two juveniles, and three infants (each of the three adult females had one infant). The social upheaval led to the group consisting of 19 or 20 members during our study periods. A genetic analysis showed that two natal infants were sired by *Nidai*, and all immigrants from the infant to the young silverback were sired by the dead silverbacks in their natal groups (*Gentil* and *Martial*), meaning that all were certainly unrelated to *Nidai* (Inoue et al. 2013; Tamura et al., in prep., Table 1). Although they were not related to the current resident silverback, no infanticide or eviction from the Nidai Group occurred. Instead, the group maintained stable membership for the length of the study period (only the immigrant young silverback left from the group).

**Table 1:** The membership of the Nidai Group

ID	Age class	Sex^a^	Father^b^	Mother^b,c^	Attribution
Mature members					
*Nidai*	SB	Male	–	–	–
*Randa*	AF (13–14*)	Female	*Gentil*	*Ovono*	Senior
*Ngou*	AF	Female	–	–	Senior
*Kojiwa*	AF	Female	–	–	Immigrant
*Ovono*	AF	Female	–	–	Immigrant
*Maria*	AF	Female	–	–	Immigrant
*Manbu*	YS (15–16*)	Male	*Gentil*	–	Immigrant
*Bengos*	BB (13–14*)	Male	*Gentil*	–	Immigrant
*Dodo*	BB (13–14*)	Male	*Gentil*	*Ngou*	Immigrant
*Sanji*	BB (12–13*)	Male	*Gentil*	*Kojiwa*	Immigrant
Immature members					
*Prince*	IF (3–4*)	Male	*Nidai*	*Ngou*	Natal
*Ranguisa*	IF (1–2*)	Female	*Nidai*	*Randa*	Natal
*Douta*	SA (8–9)	Male	*Gentil*	*Ovono*	Immigrant
*Okame*	SA (8–9*)	Male	*Martial*	*Ngou*	Immigrant
*Intsi*	SA (7–8)	Female	*Gentil*	*Kojiwa*	Immigrant
*Mituty*	JV (6–7)	male	*Gentil*	–	Immigrant
*Tsulime*	JV (5–6*)	Female	*Martial*	–	Immigrant
*Matase*	IF (4–5*)	Male	*Martial*	*Maria*	Immigrant
*Obnetu*	IF (4–5*)	Male	*Gentil*	*Ovono*	Immigrant
*Kotama*	IF (2–3)	Female	*Gentil*	*Kojiwa*	Immigrant

Numbers in parentheses indicate individual age in 2018–2019 and the asterisk refers to an estimated age. The age class is shown based on the age in 2018: SB = silverback, AF = adult female, YS = young silverback, BB = blackback, SA = subadult, JV = juvenile, IF = infant

^a^The immatures’ sex were determined by amplifications of the X–Y homologous amelogenin gene

^b^Paternity and maternity were genetically determined by Inoue et al. (2013) and Tamura et al. (in prep)

^c^Only mothers who are present in the Nidai Group appear in the table

### Definitions of terms for subject gorillas

We followed the age classification provided by Breuer et al. (2009): infants (0–4 years), juveniles (4–7.5 years), subadults (7.5–11 years for males, 7.5–10 years for females), adult females (>10 years), blackback males (11–14 years), young silverback males (14–18 years), and silverback males (>18 years old). The definition of “immature” in the context of gorilla research is ambiguous, especially for males. Some studies have labeled blackback and young silverback males mature, while other studies considered them immature, as they are sexually active (Vigilant et al. 2015) but physically and socially immature (Watts 1989). In this study, both blackbacks and young silverbacks were included in the mature category. Thus, we defined infants, juveniles, and subadults as immatures (Table 1).

As we mentioned above, we referred to the two infants sired by *Nidai* as “natal immature(s),” and we called all immigrants from infants to subadults sired by *Gentil* or *Martial* “immigrant immature(s).” In addition, we referred to adult females residing in the Nidai Group before the social upheaval (i.e., the mother of natal immatures) as “senior” female(s), and we do the newly immigrated females during the social upheaval (i.e., the mother of immigrant immatures) as “immigrant” female(s).

### Notes of subject individuals

One senior female, *Randa*, was born in the Gentil Group in 2005, a daughter of *Gentil* and *Ovono* (Inoue et al. 2013; Table 1). *Ovono* was an immigrant female having a subadult son (*Douta*) and an infant son (*Obnetu*) at the time of immigration. Beyond these three, the other two immigrant immatures from the Genti Group (*Intsi* and *Mituty*) also experienced co-residence with *Randa* in the Gentil Group for several years until *Randa*’s natal dispersal in 2014. That is, *Randa* was an acquainted full- or paternal half-sibling for the immigrant immatures from the Gentil Group, with the exception of *Kotama*, who was born in 2016 in the Gentil Group (see Table 1).

The other senior female, *Ngou*, lived in the Gentil Group and the Martial Group before arriving at the Nidai Group. She had a son *Dodo* with *Gentil* and a son *Okame* with *Martial* in the Gentil Group and the Martial Group, respectively. However, she emigrated from the former group in 2008 and from the latter group in 2014, leaving her sons behind. In 2018, *Ngou* was in the Nidai Group with a 3-year-old infant (*Prince*) fathered by *Nidai*. Then, *Dodo* and *Okame* immigrated into the Nidai Group between March and September 2018 (unpublished data). The two mother–son pairs were reunited following a decade and 4 years of separation, respectively. Despite this maternal separation, we had the impression from field observations that the pairs’ relationships were not very different from those of other pairs. Thus, we included the three individuals in our analyses concerning mother–offspring relationships.

### Behavioral observations

We conducted behavioral observations of the Nidai Group over two distinct periods: September 2018 to February 2019 and July to December 2019 (2018 and 2019). We considered that all immatures, blackbacks, and young silverbacks grew 1 year older from 2018 to 2019. We observed the members of the group by conducting group follows of more than 2 h daily. We followed the group for more than 2 h for 97 days in 2018 and 115 days in 2019. The total observation time was 601.5 h (6.2 ± 1.7 h/day) in 2018 and 743.7 h (6.5 ± 1.7 h/day) in 2019.

In this study, we set the 5 m proximity as an indicator of affiliative distance that represented social closeness between the individuals, following the technique of previous studies (e.g., Fletcher 2001; Robbins 1996; Watts 1994; Yamagiwa 1987). Once the Nidai Group was located, we began instantaneous scan sampling every 10 min (Altmann 1974), recording the individuals’ identity, any proximity within 5 m, and activity (feed-move, rest, and play) of all visible individual(s). For all visible immatures, we recorded the identity of the nearest mature members within 5 m, if available. We combined feeding and moving observations, as the two activities were not always distinguishable. In this way, we obtained 1917 and 3453 scan points in which more than one individual was visible, in 2018 and 2019, respectively (see Table S1 for the detailed numbers for the scan points).

In addition to 5 m proximity data, we intended to record affiliative interactions between the silverback and immatures, including grooming, playing, resting in contact, touching, carrying, and holding, to the end that we could assess their social relationships, as in wild mountain gorillas (Rosenbaum et al. 2015). However, these interactions were largely absent in our subject group. Brief direct contact between the silverback and an immature was observed only twice; one natal infant and one immigrant subadult female momentarily touched the silverback’s body with their hand. Thus, we used only spatial proximity and activity patterns to determine social relationships among group members.

Where we were able to observe the establishment of 5 m proximity between the silverback and an immature, we recorded the direction of approaches ad libitum. The definition of approaches was drawn from Rosenbaum et al. (2015) as follows: one individual moves into the 5 m range of another and remains within 5 m for at least 5 s. We included approaches by infants who were carried within 5 m by the mother, as was also done in Rosenbaum et al. (2011).

During group follows, the silverback sometimes exhibited charging behaviors toward the human observers (Tamura et al. 2024). Because these behaviors may have affected spatial positioning among group members, we canceled data collection during the following scan sampling. We allowed an interval of 10–20 min to elapse before resuming data collection following the charging behavior.

### Data analyses

To quantify social closeness between individuals, we estimated the amount of time that each pair spent within 5 m using the half-weight index (HWI: Bret et al. 2013; Hoppitt and Farine 2018). This index is calculated as follows: 5 mHWI = X_AB_/[X_AB_ + Y_AB_ + 0.5(Y_A_ + Y_B_)]. Here, X_AB_ is the number of scan points where individuals A and B were observed in 5 m proximity, Y_AB_ refers to the number of scan points where individual A and B were simultaneously seen but were not within 5 m, and Y_A_ and Y_B_ report the numbers of scan points where only individual A or B were observed. We could not observe all group members at each scan point, so the number of scan points was unequal across individuals. Thus, HWI was a suitable value for controlling missing individuals. We calculated 5 mHWI values for all possible pairs in each study period. In addition, we used records of the nearest mature neighbor within 5 m of each immature to calculate the nearest-neighbor HWI (NN-HWI). This index was calculated using the identical formula as 5 mHWI with X_AB_, changed to the number of scan points where mature A was the nearest neighbor of immature B. We used this index to determine socially preferred matures for each immature (e.g., Lappan 2007; McCann and Rothman 1999). Using the two types of HWI measures, we conducted the statistical analyses as detailed below.

### Statistical analyses

First, we examined the difference in social closeness with the silverback between natal immatures (*N* = 2) and immigrant immatures (*N* = 8) using 5 mHWI values. We used a generalized linear mixed model (GLMM) with a binomial distribution to fit 5 mHWI values to response variables. Immature attribution (whether natal or immigrant) and immature ages were set as explanatory variables. Immature identity was set as a random factor. We did the same analysis, adding three immigrant blackbacks and one immigrant young silverback to the immigrant immature category (i.e., the total sample size was 12 immigrants). Further, we calculated the proportion of immature-initiated approaches within 5 m of the silverback to investigate the responsibility for establishing the affiliative proximities focusing on 10 immatures (two natal and eight immigrant immatures).

Second, we examined the difference in the development of independence from the mother between natal immatures (*N* = 2) and immigrant immatures (*N* = 6, after excluding two maternal orphans) using 5 mHWI values. We used a GLMM with a binomial distribution to fit 5 mHWI values as response variables. Immature attribution and immature ages were explanatory variables. Immature identity was set as a random factor. We did the same analysis, adding the two immigrant blackbacks whose mothers were in the Nidai Group (i.e., *Sanji* and *Dodo*) to the immigrant immature category (i.e., the total sample size was eight immigrants).

Third, we examined patterns of proximity among the silverback–mother–infant triads employing the prediction that the 5 mHWI values for mother–infant pairs would be the highest, and those of the silverback–infant pairs would be higher than those for silverback–mother pairs, as observed by Rosenbaum et al. (2016b). Our samples for this analysis included two pairs of “senior female–natal infant” and three pairs of “immigrant female–immigrant infant.” We then investigated whether triad proximity patterns differed between the mother–infant pair types. We used a GLMM with a binomial distribution to fit 5 mHWI values as response variables. Mother–infant attribution (“senior female–natal infant” or “immigrant female–immigrant infant”), type of pair (mother–infant, silverback–infant, or silverback–mother), and interactions between the two predictors were set as explanatory variables. Identity of infant was set as a random factor.

Fourth, using NN-HWI values, we determined the socially preferred nonmother mature, focusing on 10 immatures (two natal and eight immigrant immatures). To determine the socially preferred matures (the candidates were 10 matures: one silverback, five adult females, one young silverback, and three blackbacks), we established a threshold NN-HWI value by calculating twice the mean NN-HWI for all mature–immature pairs, excluding mother–offspring pairs (total number of pairs: 92 pairs in 2018 and 82 pairs in 2019). With this procedure, the threshold value was set to NN-HWI < 0.062 and <0.081 in 2018 and 2019, respectively. The mature members that showed higher NN-HWI than the thresholds were considered socially preferred matures of the observed immatures in each year (this method is drawn and modified from Greenfield et al. 2022).

The fourth analysis showed that the silverback and one senior female (*Randa*) tended to be socially preferred nonmother mature for many immatures. To investigate differences in the social function of the silverback, *Randa*, and the mother for the immatures, we examined differences across the activities of the immatures when each of the three matures was the nearest neighbor. The proportion of scan points where each of the three adults was the nearest neighbor for each activity (feed-move, rest, and play) was calculated for each immature. For example, in immature A, the numbers of total scan points in feed-move, rest, and play were 300, 200, and 100, respectively. In these total points, the silverback was recorded as the nearest neighbor 60, 50, and 40 times, respectively. Here, the proportions where the silverback was the nearest neighbor for each activity were 20%, 25%, and 40%, respectively. These proportions for proximity with the silverback were calculated for all immatures, and we examined which activity showed the highest proportion when the silverback was the nearest neighbor, using GLMM. We adopted a binomial distribution to fit these proportions to response variables. The activities (feed-move, rest, and play), immature attribution (natal or immigrant), and the interaction between the predictors were set as explanatory variables. Immature identity was set as a random factor. We ran the same model for the silverback (number of samples: two natal and eight immigrant immatures), *Randa* (number of samples: one natal and eight immigrant immatures), and the mother (number of samples: two natal and six immigrant immatures), respectively. In the *Randa* model, we removed her infant offspring, *Ranguisa*, from the sample of natal immatures, as this pair was included in the mother model. In addition, for the *Randa* model, immature attribution was excluded from the explanatory variable, as only one natal immature remained after removing *Ranguisa*. In mother model, we removed two immigrant immatures who were maternal orphans (i.e., *Mituty* and *Tsulime*) from the sample.

We ran all GLMMs using the *glmer* function of the R package “lme4” (Bates et al. 2015) in R ver. 4.1.1 (R Core Team 2021). We performed Wald tests to assess whether the effects of the predictors were statistically significant. We conducted Tukey tests to perform post hoc comparisons using the R package “emmeans” (Lenth 2018). We assessed collinearity among predictors using the R package “car” (Fox and Weisberg 2011). Collinearity was not an issue because all variance inflation factors were ≤ 2.0. For all analyses, α was set at *p* ≤ 0.05.

## Results

### Social closeness between the silverback and immatures

**Fig. 1:**
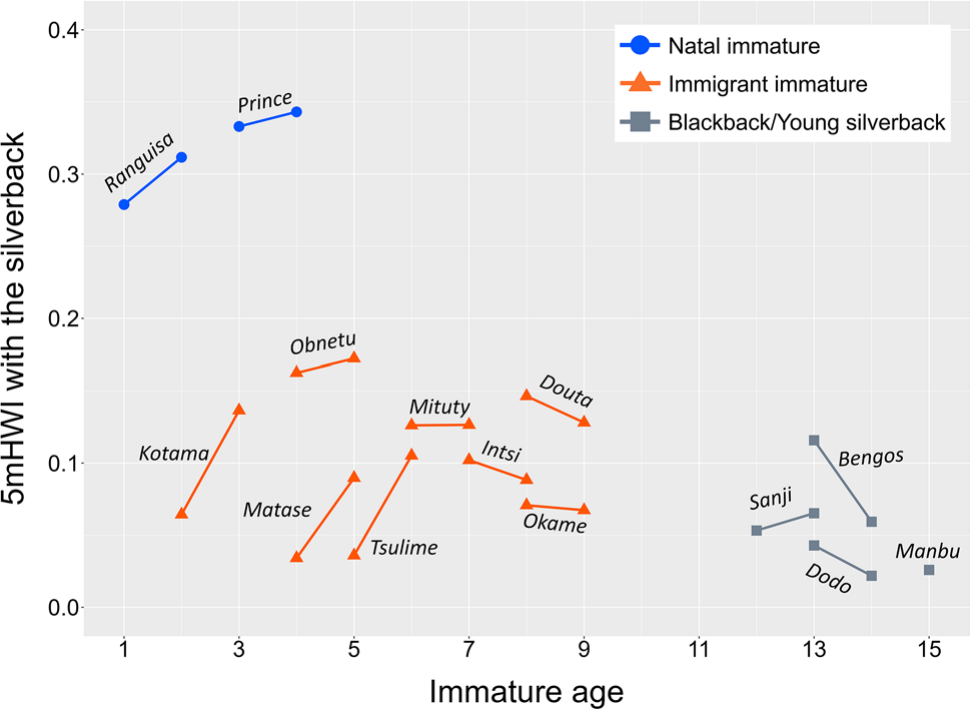
Amount of time that immatures spent within 5 m of the silverback. Short solid lines connect 5 mHWI values across different study periods (left: 2018, right: 2019) for each immature. Blue circles and orange triangles show natal and immigrant immatures, respectively. Gray squares indicate immigrant blackback and young silverback males. The individual names are given on or under the lines

Natal immatures spent significantly more time within 5 m of the silverback than immigrant immatures (GLMM: estimate = 1.35, SE = 0.28, *z* = 4.9, *p* < 0.001; Fig. 1). No linear effect was seen of immature age on 5 mHWI values involved with the silverback (GLMM: estimate = 0.06, SE = 0.04, *z* = 1.5, *p* = 0.12). The results did not change when the immigrant blackbacks (*Sanji*, *Dodo*, and *Bengos*) and young silverback (*Manbu*) were added to the immigrant immature category (effect of attribution: estimate = 1.09, SE = 0.31, *z* = 3.5, *p* < 0.001; effect of age: estimate = –0.05, SE = 0.03, *z* = –1.8, *p* = 0.07). Using HWI values, the natal immatures were estimated to spend approximately 30% of the time within 5 m of the silverback, while the values for immigrant immatures ranged only from 0.2 to 17%. Although the values for the three younger immigrant immatures (i.e., *Kotama*, *Matase*, and *Tsulime*) increased drastically between 2018 and 2019, none reached the values seen with the natal immatures (Fig. 1).

In relation to the responsibility for establishing the 5 m proximity with the silverback, all immatures initiated more than half of approaches for both study periods, except for *Intsi*, an immigrant immature, in 2018. No striking differences were seen in percentages between natal and immigrant immatures (Table 2).

**Table 2:** Responsibility for establishing 5 m proximity between the silverback and an immature

Immature ID	Immature approach %	
	2018	2019
Natal immature		
*Ranguisa*	62.9% (34/54)	77.6% (83/107)
*Prince*	65.9% (24/44)	72.3% (68/94)
Immigrant immature		
*Kotama*	75.0% (6/8)	70.4% (38/54)
*Obnetu*	59.0% (13/22)	67.2% (39/58)
*Matase*	100% (3/3)	73.1% (19/26)
*Tsulime*	80.0% (4/5)	75.7% (28/37)
*Mituty*	65.4% (17/26)	73.7% (28/38)
*Intsi*	40.0% (6/15)	69.2% (18/26)
*Douta*	85.0% (17/20)	63.0% (29/46)
*Okame*	81.8% (9/11)	78.6% (22/28)

Numerators of the ratios in parentheses are the numbers of ad libitum observations of an immature approaching within 5 m of the silverback. Denominators are the number of approaches by the silverback added to the numerator

### Development of independence from the mother

**Fig. 2:**
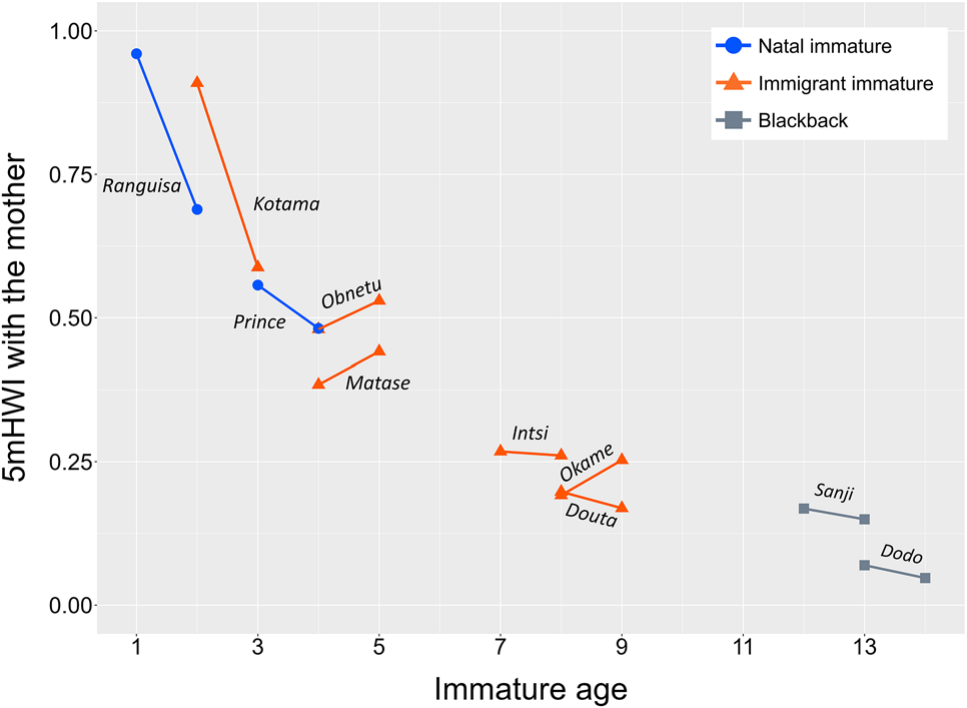
Change in the 5 m proximity pattern between mothers and immatures as immature aged. Short solid lines connect 5 mHWI values across different study periods (left: 2018, right: 2019) for each immature. Blue circles and orange triangles show natal and immigrant immatures, respectively. Gray squares indicate immigrant blackbacks. The individual names are given on or under the lines

As the immatures aged, the amount of time spent within 5 m of the mother significantly decreased (GLMM: estimate = –0.20, SE = 0.01, *z* = –17.1, *p* < 0.001; Fig. 2). There were no effects of immature attribution (natal or immigrant) on mother–offspring proximity patterns (GLMM: estimate = –0.12, SE = 0.06, *z* = –1.9, *p* = 0.059). The results did not change after adding the two immigrant blackbacks whose mothers lived in the group (*Sanji* and *Dodo*) to the sample (effect of age: estimate = –0.19, SE = 0.02, *z* = –11.7, *p* < 0.001; effect of attribution: estimate = –0.10, SE = 0.15, *z* = –0.7, *p* = 0.51).

The time spent within 5 m of the mother during the infancy (0–4 years of age) was varied in both natal and immigrant immatures (Fig. 2). The youngest natal infant *Ranguisa* spent 96.0% of her time within 5 m of the mother at the age of one. At age two in 2019, that time decreased to 68.9%. The older natal infant *Prince* spent 55.7% of his time with the mother at age three. At weaning age four the following year, that time was less than half (48.1%). The unweaned immigrant infant *Kotama* spent 90.9% of her time at the age of two, considerably longer than the same age-old natal infant *Ranguisa* (68.9%). The following year, however, that time was about the same (58.8%) as 3-year-old natal infant *Prince* (55.7%). The two immigrant infants at weaning age four (*Obnetu* and *Matase*) spent less than half of their time with their mothers (48.0% and 38.4%, respectively), as did the natal infant *Prince* (48.1%). Interestingly, however, at age five after weaning, spending time within 5 m of their mother slightly increased in both immigrant immatures (53.0% and 44.1%, respectively).

### Proximity pattern among silverback–mother–infant triads

**Fig. 3:**
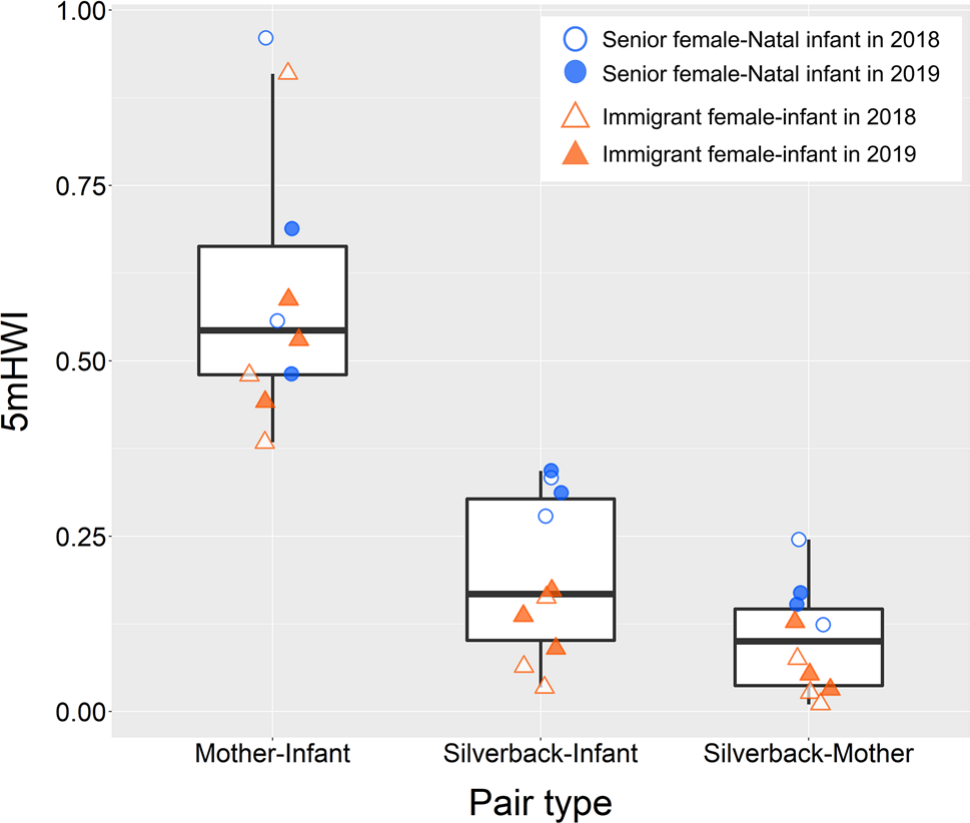
Proximity patterns among silverback–mother–infant triads, based on the 5 mHWI values. Blue circles indicate triads of the silverback, senior females, and natal infants. Orange triangles indicate triads of the silverback, immigrant females, and immigrant infants. Open and filled figures represent the values in 2018 and 2019, respectively. The boxes show the upper and lower quartiles, the line is the median, and the whiskers present the highest and lowest values, excluding outliers

Regardless of mother–infant attributions (“senior female–natal infant” or “immigrant mother–immigrant infant”), the mother–infant pairs showed the highest 5 mHWI values. In addition, silverback–infant pairs spent significantly more time within 5 m of each other than silverback–mother pairs (Table 3; Fig. 3).

**Table 3:** Effects of the interaction between the pair type and the mother–infant attribution on proximity patterns among silverback–mother–infant triads

Pair type contrast^a^	Estimate ± SE	*z* value	*p* value
Silverback-senior-natal triad^b^			
MO–IF vs. SB–IF	0.73 ± 0.05	15.6	<0.001
MO–IF vs. SB–MO	1.35 ± 0.06	24.2	<0.001
SB–IF vs. SB–MO	0.61 ± 0.06	10.5	<0.001
Silverback-immigrant-immigrant triad^c^			
MO–IF vs. SB–IF	1.52 ± 0.06	25.7	<0.001
MO–IF vs. SB–MO	2.20 ± 0.08	29.1	<0.001
SB–IF vs. SB–MO	0.68 ± 0.08	8.22	<0.001

^a^SB: silverback, MO: mother of an infant, IF: infant

^b^Triad involving senior females and natal infants

^c^Triad involving immigrant females and immigrant infants

### Socially preferred nonmother mature (Table 4; Fig. 4a, b)

In 2018, the silverback was preferred by the two natal immatures (*Ranguisa* and *Prince*) with the highest NN-HWI values exceeding the threshold. On the other hand, regarding immigrant immatures, only *Obnetu* showed the NN-HWI values of the silverback exceeding the threshold. For an immigrant immature *Matase*, the NN-HWI value of the silverback was the highest but did not exceed the threshold. Meanwhile, *Randa*, a senior female, was preferred by multiple immigrant immatures; *Obnetu*, *Mituty*, *Intsi*, and *Douta* had the highest NN-HWI values, which exceeded the threshold. Other immigrant immatures, *Kotama*, *Tsulime*, and *Okame*, showed the highest NN-HWI values for *Randa*, but did not exceed the threshold. Other than *Randa*, another senior female *Ngou* was preferred by immigrant immatures *Mituty*, and an immigrant blackback *Sanji* was preferred by a full younger sister *Intsi*, an immigrant immature.

In 2019, the top socially preferred mature for the two natal immatures (*Ranguisa* and *Prince*) was the silverback, as they showed in 2018. Regarding immigrant immatures, *Kotama* preferred the silverback with the highest NN-HWI values exceeding the threshold. For two other immigrant immatures, *Matase* and *Tsulime*, the NN-HWI values of the silverback were the highest but did not exceed the threshold. *Randa*, a senior female, was preferred by two immigrant immatures; *Mituty* and *Douta* had the highest NN-HWI values, which exceeded the threshold. For an immigrant immature *Obnetu*, the NN-HWI value of *Randa* was almost the same as that of the silverback. An immigrant blackback *Sanji* was preferred by a full younger sister *Intsi*.

**Table 4:** Top three nonmother mature members with the highest NN-HWI values in each immature in both years

Immature ID	Study period	First	Second	Third
Natal immature				
*Ranguisa* (Female infant)	2018	***Nidai* (0.087)**	*Ovono* (0.015)	*Kojiwa* (0.006)
	2019	***Nidai* (0.193)**	*Ovono* (0.053)	*Ngou* (0.040)
*Prince* (Male infant)	2018	***Nidai* (0.231)**	***Randa* (0.083)**	*Ovono* (0.014)
	2019	***Nidai* (0.233)**	*Randa* (0.058)	*Ovono* (0.035)
Immigrant immature				
*Kotama* (Female infant)	2018	*Randa* (0.039)	*Ovono* (0.021)	*Nidai* (0.020)
	2019	***Nidai* (0.090)**	*Randa* (0.060)	*Ovono* (0.050)
*Obnetu* (Male infant)	2018	***Randa* (0.135)**	***Nidai* (0.085)**	*Kojiwa* (0.058)
	2019	*Randa* (0.077)	*Nidai* (0.077)	*Sanji* (0.020)
*Matase* (Male infant)	2018	*Nidai* (0.029)	*Ovono* (0.012)	*Manbu* (0.009)
	2019	*Nidai* (0.056)	*Ovono* (0.023)	*Ngou* (0.023)
*Tsulime* (Female juvenile)	2018	*Randa* (0.040)	*Nidai* (0.031)	*Sanji* (0.014)
	2019	*Nidai* (0.075)	*Ovono* (0.073)	*Randa* (0.062)
*Mituty* (Male juvenile)	2018	***Randa* (0.280)**	***Ngou* (0.081)**	*Ovono* (0.068)
	2019	***Randa* (0.238)**	*Nidai* (0.060)	*Ovono* (0.055)
*Intsi* (Female subadult)	2018	***Randa* (0.148)**	***Sanji* (0.103)**	*Nidai* (0.054)
	2019	***Sanji* (0.085)**	*Randa* (0.069)	*Nidai* (0.048)
*Douta* (Male subadult)	2018	***Randa* (0.253)**	*Nidai* (0.068)	*Bengos* (0.040)
	2019	***Randa* (0.163)**	*Nidai* (0.075)	*Ngou* (0.041)
*Okame* (Male subadult)	2018	*Randa* (0.057)	*Nidai* (0.049)	*Kojiwa* (0.042)
	2019	*Dodo* (0.061)	*Ovono* (0.050)	*Nidai* (0.036)

The threshold value was NN-HWI < 0.062 in 2018 and <0.081 in 2019

Socially preferred matures, whose NN-HWI values exceeded the thresholds, are bolded

**Fig. 4:**
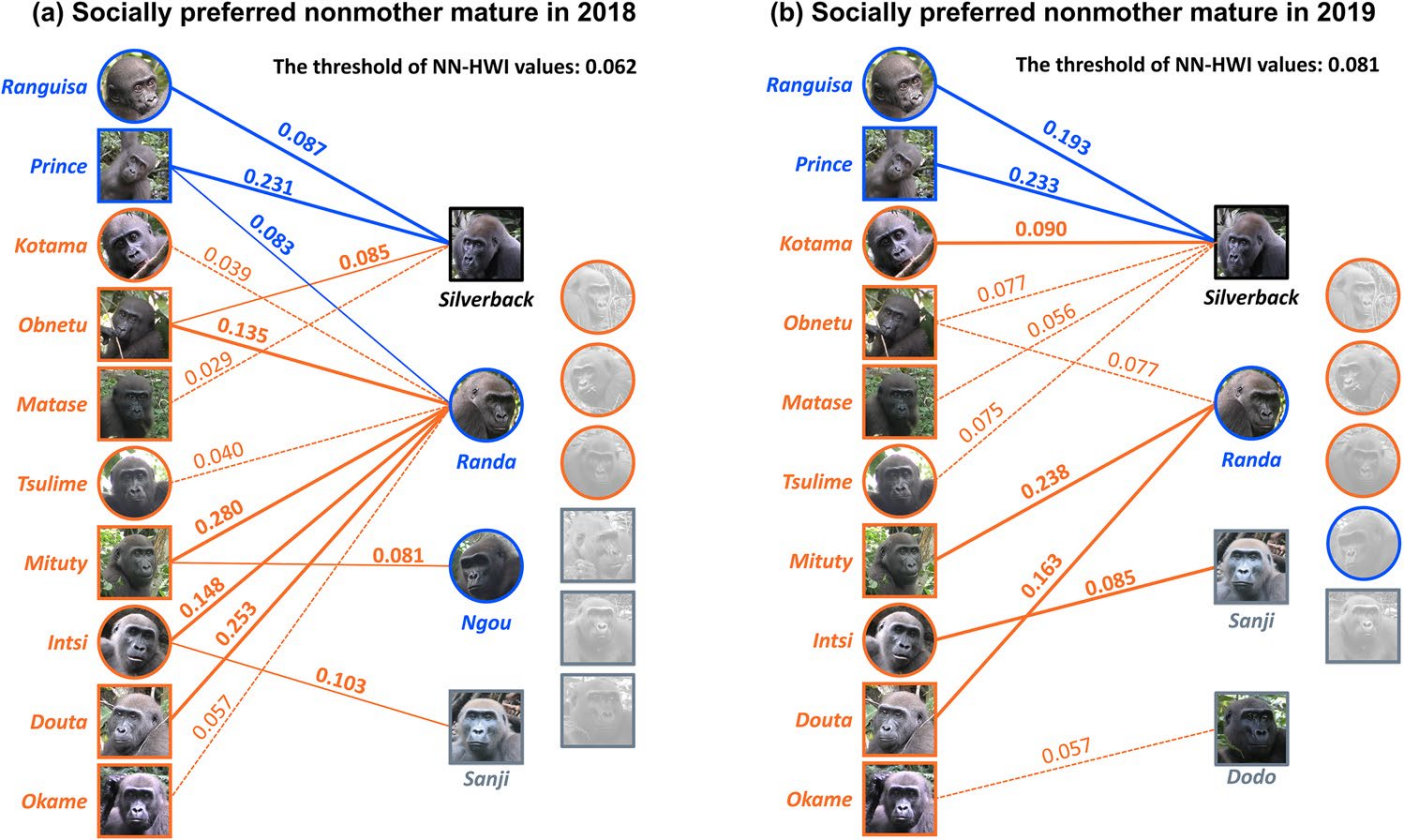
Diagrammatic illustration of the socially preferred nonmother matures for each immature in **a** 2018 and **b** 2019. The images on the left line are immatures and those on the middle and right lines are candidate matures. Images with blue frames indicate natal immatures and senior females, and those with orange frames do immigrant immatures and immigrant females. Images with gray frames are immigrant blackback and young silverback males. The silverback is framed in black color. Square and round shapes indicate male and female individuals, respectively. Bold solid lines mean a socially preferred mature with the highest NN-HWI value exceeding a threshold. Thin solid lines mean a socially preferred mature with the second highest NN-HWI value exceeding a threshold. Thin dashed lines mean a mature with the highest NN-HWI value that does not exceed a threshold. Candidate matures shown in the grayscale image are individuals with no NN-HWI values fitting the above criteria. The individual names are given on the left side or below the images. The NN-HWI values are given on the line connecting an immatures and a preferred mature

### Immature activities when the nearest neighbor was the silverback, *Randa*, or mother

**Fig. 5:**
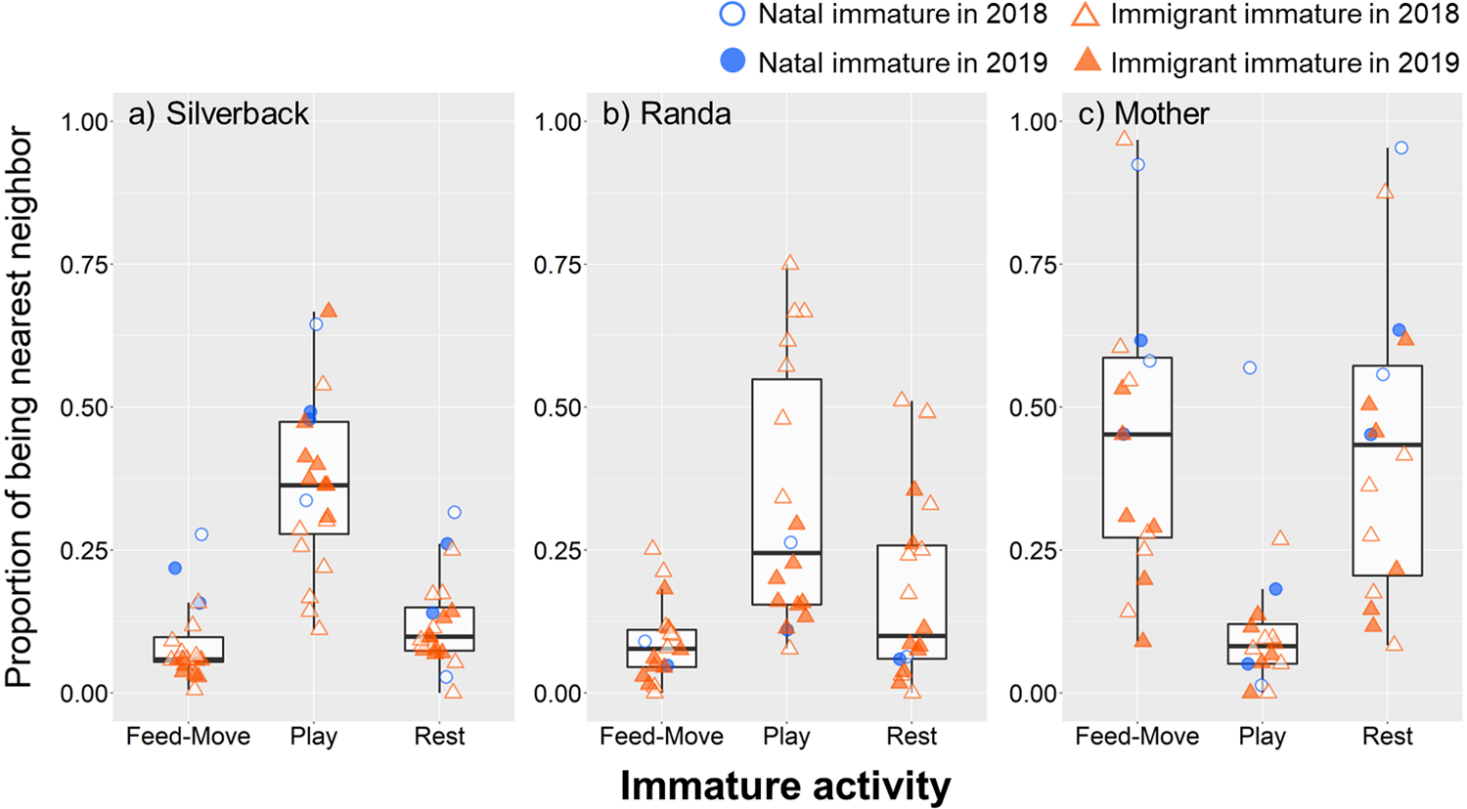
Immatures’ activities when **a** the silverback, **b** *Randa*, or **c** the mother was the nearest neighbor. Blue circles and orange triangles show natal and immigrant immatures, respectively. Open and filled figures represent the values in 2018 and 2019, respectively. The boxes show the upper and lower quartiles, the line is the median, and the whiskers present the highest and lowest values, excluding outliers

The above analyses indicate that the immatures were often near the silverback, *Randa*, or their mothers. We then examined the differences in the proportion of immature activity for the cases where these matures were the nearest neighbor. Where the silverback was the nearest neighbor, the proportion of playing was significantly higher than those for the other activities, regardless of immature attribution (Table 5; Fig. 5a). The same tendency was found in *Randa*, where the immatures played with each other more often than they fed, moved, or rested around her (Table 6; Fig. 5b). By contrast, when the mother was the nearest neighbor, the immature activity was largely different from the case of the silverback and *Randa*, in that the proportion of playing was significantly lower than those for feeding–moving and resting in both natal and immigrant immatures (Table 7; Fig. 5c).

**Table 5:** Effects of the interaction between activities and immature attributions on proportions of *Nidai* being the nearest neighbor

Activity contrast	Estimate ± SE	*z* value	*p* value
Natal immature			
Play vs. feed-move	1.07 ± 0.11	9.82	<0.001
Play vs. rest	1.07 ± 0.12	8.87	<0.001
Feed-move vs. rest	0.001 ± 0.11	0.01	1.00
Immigrant immature			
Play vs. feed-move	1.92 ± 0.11	17.3	<0.001
Play vs. rest	1.28 ± 0.11	11.5	<0.001
Feed-move vs. rest	–0.64 ± 0.10	–6.77	<0.001

**Table 6:** Effects of the activities on proportions of *Randa* being the nearest neighbor

Activity contrast	Estimate ± SE	*z* value	*p* value
Play vs. feed-move	1.34 ± 0.10	13.7	<0.001
Play vs. rest	0.72 ± 0.10	7.34	<0.001
Feed-move vs. rest	–0.62 ± 0.07	–8.80	<0.001

**Table 7:** Effects of the interaction between activities and immature attributions on proportions of *Mother* being the nearest neighbor

Activity contrast	Estimate ± SE	*z* value	*p* value
Natal immature			
Play vs. feed-move	–1.17 ± 0.12	–9.50	<0.001
Play vs. rest	–1.17 ± 0.13	–9.24	<0.001
Feed-move vs. rest	0.001 ± 0.07	0.01	1.00
Immigrant immature			
Play vs. feed-move	–1.54 ± 0.16	–9.46	<0.001
Play vs. rest	–1.49 ± 0.17	–9.02	<0.001
Feed-move vs. rest	0.05 ± 0.06	0.75	0.98

## Discussion

Natal immatures spent a greater proportion of time within 5 m of the resident silverback than immigrant immatures. A similar pattern of social closeness between a resident male and immatures has also been reported in other primates, such as in captive western lowland gorillas (Enciso et al. 1999), equatorial sakis (Di Fiore et al. 2007), Azara’s owl monkeys (*A. azarai*) (Huck and Fernandez-Duque 2012b), and the stepfathers and stepchildren of human hunter–gatherers (Marlowe 1999). Meanwhile, the 5 mHWI values for the three younger immigrant immatures sharply increased between 2018 and 2019, with inference that 1 year of co-residence with the silverback could somewhat improve social closeness. Similarly, Yamagiwa (2001) reported that immigrant immatures in a group of wild eastern lowland gorillas (*G. b. graueri*) began to play with an unrelated silverback in a new group after 6 months of co-residence, after an initial period of avoidance. This evidence indicates that kinship may not be a primary determinant, but rather familiarity, which is created by the experience of co-residence, may have a larger effect on the social closeness between a resident silverback and immatures in gorillas. However, in the current case, the 5 mHWI values for the three immigrant immatures in 2019 remained considerably below those of natal immatures. This result suggests that 1 year of co-residence may be insufficient to develop comparable familiarity with natal immatures.

In theory, the unfamiliar social condition of co-residence with an unrelated silverback could delay immatures from achieving independence from their mothers. Indeed, a juvenile of wild pair-bonded equatorial sakis increased proximity with its mother after the replacement of the resident male (Di Fiore et al. 2007). Here, however, mother–offspring proximity significantly decreased with immature ages and there was no significant difference between natal and immigrant immatures. Our analysis found that the amount of time that the immatures spent within 5 m of their mothers was approximately 50% around 4 years old. In mountain gorillas, this occurred around 2.5 years old (Fletcher 2001). Taking into account the slower development of western lowland gorillas than mountain gorillas (Breuer et al. 2009), the immigrant immatures in the Nidai Group seemed to follow normal developmental patterns in terms of independence from the mother. This indicates that co-residence with the nonfather silverback did not lead to any delayed independence from the mother in our observations.

In the genus *Gorilla*, the silverback in a group is well known to be the spatial focus for immatures during and following independence from the mother (Fossey 1979; Rosenbaum et al. 2011; Yamagiwa 1983). Likewise, in this study, the silverback was the socially preferred nonmother mature for the two natal immatures. Identical results were seen in wild mountain gorillas (Stewart 2001) and captive western lowland gorillas (McCann and Rothman 1999). On the other hand, our results indicated that certain immigrant immatures (*Obnetu*, *Mituty*, *Intsi*, and *Douta*) socially preferred a senior female *Randa* to the silverback. As noted, she was the full or paternal half-sister of these immigrant immatures, and they had previously co-resided for several years in their natal Gentil Group. Contrarily, the other immigrant immatures, who had not lived together with *Randa* in the same group before (i.e., *Kotama*, *Matase*, *Tsulime*, and *Okame*), did not show NN-HWI values exceeding the thresholds with *Randa*. This result implies that not only siblingship but also familiarity through co-residence experiences can affect the choice of an alternative social partner. Such effects of siblingship and familiarity on affiliative relationships were also found in mountain gorillas (Grebe et al. 2022). Still, in many cases, *Randa* was the first or second top nearest neighbor of these immatures. These results suggest that, in western lowland gorillas, an adult female can be an alternative spatial focus for immatures during or after independence from the mother, where the current resident silverback is not a favorable figure.

When both natal and immigrant immatures engaged in play, the silverback or *Randa* were more likely to be their nearest neighbor than they performed other activities. Contrarily, they were less likely to play when the mother was the nearest neighbor. This supports the idea that *Randa* was an alternative figure to the silverback for immatures, not their mother. This marks the first evidence that an adult female can be a play space for immature gorillas—following a social role that is often attributed to resident silverbacks (Fletcher 1994; Fossey 1979; Schaller 1963; Yamagiwa 1983). In the Nidai Group, proximity to *Randa* could be the ideal social environment for all immatures to engage in playing, as both natal and immigrant immatures could gather without hesitation. Social play is thought to strengthen social bonds among immatures in gorillas (Létang et al. 2021), as observed in chimpanzees (*Pan troglodytes*) (Shimada and Sueur 2014) and Japanese macaques (*Macaca fuscata*) (Shimada and Sueur 2018). These social bonds among immatures, created through playing around *Randa*, could lead the immigrant immatures to play with natal immatures around the silverback. This process could have gradually established social closeness between the silverback and immigrant immatures. This idea seems to be consistent with our finding that the number of immigrant immatures who had the highest NN-HWI values with the silverback increased in 2019. Altogether, in the case of the Nidai Group, the presence of *Randa* may have played an important role in the assimilation of immigrant immatures in the non-natal group.

While *Randa*’s role in the assimilation of immigrant immatures is intriguing, it should also be noted that immigrant immatures did not peripheralize within the Nidai Group; instead, their day-to-day close proximities with the silverback were seen without any agonistic interactions. This is in contrast with other primates living in one-male groups, where the new male often shows hostility toward unrelated immatures (Ohsawa 2003; Steenbeek 1996; Steenbeek et al. 2000; Watts 1989). We found that 5 m proximities between the silverback and immigrant immatures were more often initiated by the immatures. This pattern is consistent with the silverback–immature pairs of mountain gorillas (Rosenbaum et al. 2011; Stewart 2001) and captive western lowland gorillas (Enciso et al. 1999; McCann and Rothman 1999). Further, we found that immigrant infants spent more time near the silverback than their mothers did, indicating that they sometimes established close proximity with the silverback, away from their mothers. These facts suggest that social closeness between the silverback and immigrant immatures may be primarily determined by whether the immatures seek proximity to the silverback. From the perspective of the silverback, the presence of immigrant immatures within his group and even nearby would be costless, as he never showed affiliative interactions toward any immatures. Gettler et al. (2020) argued that when females prefer males who are affiliative to offspring and caring behaviors are low-cost, males can be tolerant of any immature, even when the paternity is uncertain. In our case, if *Nidai* can enjoy reproduction privileges with immigrant females by simply exhibiting non-hostile attitudes toward their offspring, he might be willing to reside with them in his group. In mountain gorillas, it has been reported that silverbacks who show high affiliations with infants, judged by grooming and resting in contact, achieve high reproductive success (Rosenbaum et al. 2018). In our case of wild western lowland gorillas, simply allowing immigrant immatures to remain in the group may be a reproductive tactic for a silverback.

In a one-male society, where group disintegration is not uncommon, there should be some systems in place to prevent fatherless immatures from being abandoned. Otherwise, each time a group disintegrates, widowed females incur a large cost of losing their young. One possible tactic of gorilla females to prevent such costs has been reported from the Mbeli gorillas: leaving silverbacks who are near the end of their tenure or closer to their death (Manguette et al. 2020). The current study provided another possible way: fatherless immatures and their mothers choose an adjacent one-male group where a leading silverback with high tolerance toward unrelated immatures and an acquainted adult female are present. From the standpoint of silverbacks, infanticide and rejection of unrelated immatures are not the only reproductive tactics; acceptance of unrelated immatures may bring them fitness benefits. Further studies will demonstrate the remarkable flexibility of social relationships in wild western lowland gorillas that enable the maintenance of the prevalent one-male social system.

### Supplementary Information

Below is the link to the electronic supplementary material.

Supplementary file1 (DOCX 23 KB)

## Data Availability

The data that support the findings of this study are available from the corresponding author upon reasonable request.
