# Supplementary material for: Does kinship with the silverback matter? Intragroup social relationships of immature wild western lowland gorillas after social upheaval
Source: Primates. 2024 Aug 10;65(5):397–410. doi: 10.1007/s10329-024-01149-1 (PMC11335836; doi:10.1007/s10329-024-01149-1)
Supplement: Supplementary file 1 — Supplementary file1 (DOCX 23 KB) [file 10329_2024_1149_MOESM1_ESM.docx]

|  | **2018** | | | |  | **2019** | | | | |
| --- | --- | --- | --- | --- | --- | --- | --- | --- | --- | --- |
| **ID** | **Feed-move** | **Rest** | **Play** | **Total** |  | **Feed-move** | | **Rest** | **Play** | **Total** |
| **Mature members** | | |  |  |  |  |  |  |  |  |
| *Nidai* | 395 | 310 | – | 705 |  | 600 | | 543 | – | 1143 |
| *Ngou* | 187 | 74 | – | 261 |  | 535 | | 238 | – | 773 |
| *Randa* | 349 | 276 | – | 625 |  | 528 | | 396 | – | 924 |
| *Kojiwa* | 192 | 79 | – | 271 |  | 346 | | 221 | – | 567 |
| *Ovono* | 200 | 70 | – | 270 |  | 408 | | 251 | – | 659 |
| *Maria* | 52 | 15 | – | 67 |  | 193 | | 120 | – | 313 |
| *Manbu* | 76 | 68 | – | 144 |  | – | | – | – | – |
| *Bengos* | 201 | 113 | – | 314 |  | 343 | | 200 | – | 543 |
| *Dodo* | 131 | 97 | – | 228 |  | 236 | | 177 | – | 413 |
| *Sanji* | 206 | 141 | – | 347 |  | 356 | | 280 | – | 636 |
| **Immature members** | |  |  |  |  |  |  |  |  |  |
| *Prince* | 155 | 79 | 76 | 310 |  | 485 | | 267 | 116 | 868 |
| *Ranguisa* | 318 | 217 | 95 | 630 |  | 500 | | 375 | 163 | 1038 |
| *Douta* | 240 | 108 | 39 | 387 |  | 516 | | 313 | 26 | 855 |
| *Okame* | 68 | 40 | 7 | 115 |  | 367 | | 198 | 15 | 580 |
| *Intsi* | 216 | 97 | 21 | 334 |  | 487 | | 274 | 22 | 783 |
| *Mituty* | 275 | 94 | 36 | 405 |  | 534 | | 288 | 44 | 866 |
| *Tsulime* | 37 | 29 | 6 | 72 |  | 474 | | 229 | 18 | 721 |
| *Matase* | 33 | 12 | 13 | 58 |  | 273 | | 170 | 19 | 462 |
| *Obnetu* | 139 | 69 | 73 | 281 |  | 414 | | 249 | 75 | 738 |
| *Kotama* | 185 | 64 | 41 | 290 |  | 388 | | 272 | 117 | 777 |

Table S1 Details of scan points for each member collected in 2018 and 2019.
